# Poly[[μ_10_-2,3-bis(carboxymethyl)butanedioato]disodium]

**DOI:** 10.1107/S1600536810040857

**Published:** 2010-10-20

**Authors:** Jiang Wu, Hong-lin Zhu

**Affiliations:** a State Key Laboratory Base of Novel Functional Materials and Preparation Science, Center of Applied Solid State Chemistry Research, Ningbo University, Ningbo, Zhejiang, 315211, People’s Republic of China

## Abstract

The asymmetric unit of the title compound, [Na_2_(C_8_H_8_O_8_)]_*n*_, contains one Na^+^ ion and half of a 2,3-bis(carboxymethyl)butanedioate (H_2_BTC^2−^) dianion, which lies on a center of symmetry. The dianion exhibits a μ_10_-bridging mode. Each Na atom lies in a NaO_6_ octa­hedron defined by six O atoms from five dianions. Adjacent NaO_6_ octa­hedra share a common O—O edge, generating a biocta­hedron; adjacent biocta­hedra are O—O edge-connected to one another, building up a chain along [001]. The chains are connected by adjacent H_2_BTC^2−^ anions into a three-dimensional framework. The structure is further stabilized by O—H⋯O hydrogen bonds.

## Related literature

For related structures, see: Delgado *et al.* (2007 ▶); Liu *et al.* (2008 ▶); Wang *et al.* (2005 ▶); Zheng *et al.* (2004 ▶); Zhu & Zheng (2010 ▶).
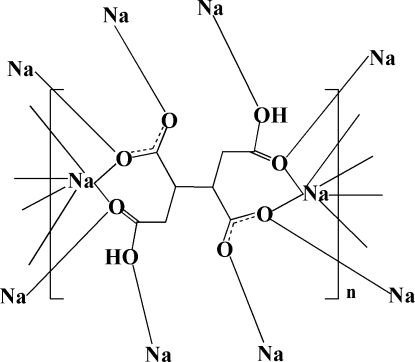


## Experimental

### 

#### Crystal data

[Na_2_(C_8_H_8_O_8_)]
*M* *_r_* = 278.12
Orthorhombic, 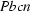

*a* = 8.9053 (18) Å
*b* = 8.6395 (17) Å
*c* = 12.527 (3) Å
*V* = 963.8 (3) Å^3^
*Z* = 4
Mo *K*α radiation
μ = 0.24 mm^−1^
*T* = 293 K
0.44 × 0.36 × 0.32 mm

#### Data collection

Rigaku R-AXIS RAPID diffractometer
Absorption correction: multi-scan (*ABSCOR*; Higashi, 1995 ▶) *T*_min_ = 0.900, *T*_max_ = 0.925
8610 measured reflections
1097 independent reflections
1000 reflections with *I* > 2σ(*I*)
*R*_int_ = 0.021

#### Refinement

*R*[*F* ^2^ > 2σ(*F* ^2^)] = 0.038
*wR*(*F* ^2^) = 0.111
*S* = 1.10
1097 reflections
86 parameters
1 restraint
H atoms treated by a mixture of independent and constrained refinement
Δρ_max_ = 0.43 e Å^−3^
Δρ_min_ = −0.22 e Å^−3^

### 

Data collection: *RAPID-AUTO* (Rigaku, 1998 ▶); cell refinement: *RAPID-AUTO*; data reduction: *CrystalStructure* (Rigaku/MSC, 2004 ▶); program(s) used to solve structure: *SHELXS97* (Sheldrick, 2008 ▶); program(s) used to refine structure: *SHELXL97* (Sheldrick, 2008 ▶); molecular graphics: *SHELXTL* (Sheldrick, 2008 ▶); software used to prepare material for publication: *SHELXL97*.

## Supplementary Material

Crystal structure: contains datablocks ptcLa, I. DOI: 10.1107/S1600536810040857/ng5044sup1.cif

Structure factors: contains datablocks I. DOI: 10.1107/S1600536810040857/ng5044Isup2.hkl

Additional supplementary materials:  crystallographic information; 3D view; checkCIF report

## Figures and Tables

**Table 1:** Hydrogen-bond geometry (Å, °)

*D*—H⋯*A*	*D*—H	H⋯*A*	*D*⋯*A*	*D*—H⋯*A*
O2—H2*C*⋯O4^i^	0.85 (2)	1.67 (3)	2.5097 (18)	177 (2)

Symmetry code: (i) 
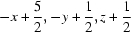
.

**Fig. 1.**
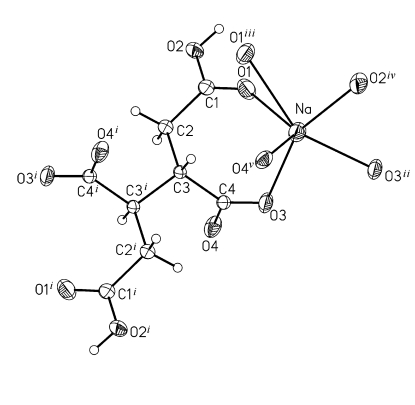
The content of asymmetric unit showing the atomic numbering and 45% probability dispalcement ellipsoids.[Symmetry codes: (i) -x + 2, -y + 1, -z + 1. (ii) -x + 2, -y, -z + 1. (iii) -x + 2, y, -z + 1.5. (iv) x - 1/2,y - 1/2, -z + 1.5. (v) x - 1/2, -y + 1/2, -z + 1.]

**Fig. 2.**
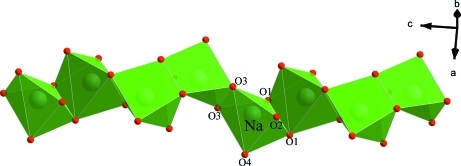
The one-dimensional sodium-oxide chains with the common edges O1—O1 and O3—O3.

**Fig. 3.**
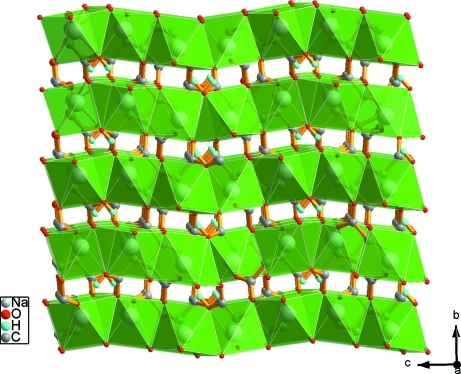
The three-dimensional metal-organic framework in the title compound.

## supplementary crystallographic information

### Comment

Recently, the aliphatic multi-carboxylic acids have attractived considerable attention due to both its conformational flexibility and a variety of coordination fashions (Wang *et al.*, 2005; Zheng *et al.*, 2004).The butane-1,2,3,4-tetracarboxylic acid (H_4_BTC) ligand possesses four ionizable protons that can be removed gradually to form a series of deprotonated anions such as H_3_BTC^-^, H_2_BTC^2-^, HBTC^3-^, BTC^4-^, which have allowed the preparation of a variety of complexes with differents metals (Delgado *et al.*, 2007; Liu *et al.*, 2008; Zhu *et al.*, 2010). In this contribution, we report the synthesis and crystal structure of the title compound.

The asymmetric unit of the title compound contains one Na^+^ ion and half a H_2_BTC^2-^ anion(Figure 1). The H_2_BTC^2-^ligand is diprotonated, which is crystallographically imposed by symmetry of center with inversion centers at the midpoints of the central C3—C3*^i^* bond with the Wyckoff 4*b* site. Each H_2_BTC^2-^ anions coordinate ten sodium ions through eight carboxyl oxygen atoms. The carboxylate group and carboxylic group all coordinates to two metal atoms in a *syn*/*anti* µ_2_η^2^ bridging fashion, and two seven-membered chelating rings are concomitantly formed. Each Na atom is in a distorted octahedra NaO_6_ gemetry defined by six O atoms from five H_2_BTC^2-^ ligands, the Na—O contact distances are all within the normal ranges. The adjacent two NaO_6_ octahedra are fused *via* common edge O1—O1 and O3—O3, generating a one-dimensional sodium-oxide chains (Figure 2), and the resulting chains are further interlinked by H_2_BTC^2-^ anions into three-dimensional frameworks (Figure 3).

### Experimental

All chemicals were obtained from commerical sources and were used as obtained. NaOH (0.079 g, 1.98 mmol) was added to a stirred mixture solution of butane-1,2,3,4-tetracarboxylic acid (0.1173 g, 0.50 mmol) in 10 ml H_2_O and 10 ml me thanol, and the resulting mixture was stirred for 5 min. Colorless crystals were obtained from the solution (pH = 7.13) after standing at room temperature for five weeks.

### Refinement

H atoms bonded to C atoms were palced in geometrically calculated position and were refined using a riding model, with *U*_iso_(H) = 1.2 *U*_eq_(C). H atoms attached to O atoms were found in a difference Fourier synthesis and refined with the O—H distance restranied to 0.83 (1) Å.

### Crystal data

**Table:**

[Na_2_(C_8_H_8_O_8_)]	*F*(000) = 568
*M**_r_* = 278.12	*D*_x_ = 1.917 Mg m^−^^3^
Orthorhombic, *P**b**c**n*	Mo *K*α radiation, λ = 0.71073 Å
Hall symbol: -P 2n 2ab	Cell parameters from 7116 reflections
*a* = 8.9053 (18) Å	θ = 3.3–27.4°
*b* = 8.6395 (17) Å	µ = 0.24 mm^−^^1^
*c* = 12.527 (3) Å	*T* = 293 K
*V* = 963.8 (3) Å^3^	Block, colorless
*Z* = 4	0.44 × 0.36 × 0.32 mm

### Data collection

**Table:**

Rigaku R-AXIS RAPID diffractometer	1097 independent reflections
Radiation source: fine-focus sealed tube	1000 reflections with *I* > 2σ(*I*)
graphite	*R*_int_ = 0.021
Detector resolution: 0 pixels mm^-1^	θ_max_ = 27.4°, θ_min_ = 3.3°
ω scan	*h* = −11→11
Absorption correction: multi-scan (*ABSCOR*; Higashi, 1995)	*k* = −11→11
*T*_min_ = 0.900, *T*_max_ = 0.925	*l* = −16→16
8610 measured reflections	

### Refinement

**Table:**

Refinement on *F*^2^	Primary atom site location: structure-invariant direct methods
Least-squares matrix: full	Secondary atom site location: difference Fourier map
*R*[*F*^2^ > 2σ(*F*^2^)] = 0.038	Hydrogen site location: inferred from neighbouring sites
*wR*(*F*^2^) = 0.111	H atoms treated by a mixture of independent and constrained refinement
*S* = 1.10	*w* = 1/[σ^2^(*F*_o_^2^) + (0.0658*P*)^2^ + 0.5154*P*] where *P* = (*F*_o_^2^ + 2*F*_c_^2^)/3
1097 reflections	(Δ/σ)_max_ < 0.001
86 parameters	Δρ_max_ = 0.43 e Å^−^^3^
1 restraint	Δρ_min_ = −0.22 e Å^−^^3^

### Special details

**Table:**

Geometry. All e.s.d.'s (except the e.s.d. in the dihedral angle between two l.s. planes) are estimated using the full covariance matrix. The cell e.s.d.'s are taken into account individually in the estimation of e.s.d.'s in distances, angles and torsion angles; correlations between e.s.d.'s in cell parameters are only used when they are defined by crystal symmetry. An approximate (isotropic) treatment of cell e.s.d.'s is used for estimating e.s.d.'s involving l.s. planes.
Refinement. Refinement of *F*^2^ against ALL reflections. The weighted *R*-factor *wR* and goodness of fit *S* are based on *F*^2^, conventional *R*-factors *R* are based on *F*, with *F* set to zero for negative *F*^2^. The threshold expression of *F*^2^ > σ(*F*^2^) is used only for calculating *R*-factors(gt) *etc*. and is not relevant to the choice of reflections for refinement. *R*-factors based on *F*^2^ are statistically about twice as large as those based on *F*, and *R*- factors based on ALL data will be even larger.

### Fractional atomic coordinates and isotropic or equivalent isotropic displacement parameters (Å^2^)

**Table:**

	*x*	*y*	*z*	*U*_iso_*/*U*_eq_	
Na	0.91916 (8)	0.06655 (8)	0.62976 (5)	0.0292 (2)	
O1	1.12521 (17)	0.21959 (14)	0.69249 (10)	0.0391 (4)	
O2	1.30500 (16)	0.37558 (13)	0.75029 (10)	0.0325 (3)	
C1	1.19167 (19)	0.34236 (18)	0.68752 (12)	0.0243 (3)	
C2	1.15019 (18)	0.46906 (17)	0.61037 (11)	0.0219 (3)	
H2A	1.2386	0.4963	0.5692	0.026*	
H2B	1.1207	0.5598	0.6508	0.026*	
C3	1.02287 (16)	0.42768 (14)	0.53277 (10)	0.0161 (3)	
H3A	0.9358	0.3927	0.5741	0.019*	
C4	1.07185 (16)	0.29671 (16)	0.45770 (11)	0.0177 (3)	
O3	1.01257 (15)	0.16760 (12)	0.46431 (9)	0.0300 (3)	
O4	1.17325 (15)	0.33112 (14)	0.39020 (10)	0.0300 (3)	
H2C	1.310 (4)	0.308 (3)	0.7989 (19)	0.088 (11)*	

### Atomic displacement parameters (Å^2^)

**Table:**

	*U*^11^	*U*^22^	*U*^33^	*U*^12^	*U*^13^	*U*^23^
Na	0.0336 (4)	0.0244 (4)	0.0296 (4)	−0.0038 (3)	0.0023 (3)	−0.0031 (2)
O1	0.0481 (8)	0.0278 (7)	0.0415 (7)	−0.0062 (6)	−0.0172 (6)	0.0104 (5)
O2	0.0432 (7)	0.0262 (6)	0.0282 (6)	0.0011 (5)	−0.0177 (5)	0.0041 (5)
C1	0.0305 (8)	0.0216 (7)	0.0208 (7)	0.0046 (6)	−0.0054 (6)	−0.0007 (5)
C2	0.0278 (7)	0.0185 (7)	0.0194 (7)	0.0020 (6)	−0.0054 (6)	0.0003 (5)
C3	0.0208 (7)	0.0137 (6)	0.0138 (6)	0.0032 (5)	0.0005 (5)	0.0002 (5)
C4	0.0212 (7)	0.0160 (6)	0.0159 (6)	0.0039 (5)	−0.0012 (5)	−0.0008 (5)
O3	0.0448 (7)	0.0166 (5)	0.0286 (6)	−0.0050 (5)	0.0081 (5)	−0.0041 (4)
O4	0.0340 (6)	0.0247 (6)	0.0314 (6)	−0.0026 (5)	0.0148 (5)	−0.0082 (5)

### Geometric parameters (Å, °)

**Table:**

Na—O4^i^	2.3748 (15)	O2—H2C	0.843 (10)
Na—O1	2.3943 (15)	C1—C2	1.506 (2)
Na—O3	2.3978 (13)	C2—C3	1.536 (2)
Na—O3^ii^	2.4188 (13)	C2—H2A	0.9700
Na—O2^iii^	2.4522 (14)	C2—H2B	0.9700
Na—O1^iv^	2.6196 (15)	C3—C4	1.5346 (18)
Na—Na^iv^	3.3388 (14)	C3—C3^vi^	1.550 (2)
Na—Na^ii^	3.7369 (14)	C3—H3A	0.9800
O1—C1	1.216 (2)	C4—O3	1.2368 (18)
O1—Na^iv^	2.6196 (15)	C4—O4	1.2723 (19)
O2—C1	1.311 (2)	O3—Na^ii^	2.4188 (13)
O2—Na^v^	2.4522 (14)	O4—Na^vii^	2.3748 (15)
			
O4^i^—Na—O1	122.39 (5)	C1—O1—Na	146.90 (11)
O4^i^—Na—O3	95.36 (5)	C1—O1—Na^iv^	123.86 (11)
O1—Na—O3	79.44 (5)	Na—O1—Na^iv^	83.38 (5)
O4^i^—Na—O3^ii^	119.46 (5)	C1—O2—Na^v^	146.49 (11)
O1—Na—O3^ii^	115.41 (6)	C1—O2—H2C	109 (2)
O3—Na—O3^ii^	78.24 (5)	Na^v^—O2—H2C	90 (2)
O4^i^—Na—O2^iii^	86.14 (5)	O1—C1—O2	122.32 (14)
O1—Na—O2^iii^	119.22 (5)	O1—C1—C2	123.18 (14)
O3—Na—O2^iii^	156.69 (5)	O2—C1—C2	114.49 (14)
O3^ii^—Na—O2^iii^	80.80 (5)	C1—C2—C3	114.71 (13)
O4^i^—Na—O1^iv^	76.26 (5)	C1—C2—H2A	108.6
O1—Na—O1^iv^	63.76 (7)	C3—C2—H2A	108.6
O3—Na—O1^iv^	127.10 (5)	C1—C2—H2B	108.6
O3^ii^—Na—O1^iv^	150.95 (5)	C3—C2—H2B	108.6
O2^iii^—Na—O1^iv^	75.89 (5)	H2A—C2—H2B	107.6
O4^i^—Na—Na^iv^	119.52 (4)	C4—C3—C2	110.47 (11)
O1—Na—Na^iv^	51.20 (4)	C4—C3—C3^vi^	110.16 (13)
O3—Na—Na^iv^	129.07 (5)	C2—C3—C3^vi^	109.98 (14)
O3^ii^—Na—Na^iv^	109.34 (4)	C4—C3—H3A	108.7
O2^iii^—Na—Na^iv^	68.03 (4)	C2—C3—H3A	108.7
O1^iv^—Na—Na^iv^	45.42 (3)	C3^vi^—C3—H3A	108.7
O4^i^—Na—Na^ii^	112.22 (4)	O3—C4—O4	123.93 (13)
O1—Na—Na^ii^	99.22 (5)	O3—C4—C3	120.17 (12)
O3—Na—Na^ii^	39.32 (3)	O4—C4—C3	115.89 (12)
O3^ii^—Na—Na^ii^	38.92 (3)	C4—O3—Na	122.30 (9)
O2^iii^—Na—Na^ii^	119.06 (4)	C4—O3—Na^ii^	127.90 (10)
O1^iv^—Na—Na^ii^	162.35 (5)	Na—O3—Na^ii^	101.76 (5)
Na^iv^—Na—Na^ii^	128.23 (4)	C4—O4—Na^vii^	144.24 (11)

Symmetry codes: (i) *x*−1/2, −*y*+1/2, −*z*+1; (ii) −*x*+2, −*y*, −*z*+1; (iii) *x*−1/2, *y*−1/2, −*z*+3/2; (iv) −*x*+2, *y*, −*z*+3/2; (v) *x*+1/2, *y*+1/2, −*z*+3/2; (vi) −*x*+2, −*y*+1, −*z*+1; (vii) *x*+1/2, −*y*+1/2, −*z*+1.

### Hydrogen-bond geometry (Å, °)

**Table:**

*D*—H···*A*	*D*—H	H···*A*	*D*···*A*	*D*—H···*A*
O2—H2C···O4^viii^	0.85 (2)	1.67 (3)	2.5097 (18)	177 (2)

Symmetry codes: (viii) −*x*+5/2, −*y*+1/2, *z*+1/2.
